# Commentary on Structured chaos shapes spike-response noise entropy in balanced neural networks, by Lajoie, Thivierge, and Shea-Brown

**DOI:** 10.3389/fncom.2015.00023

**Published:** 2015-03-10

**Authors:** Peter J. Thomas

**Affiliations:** Department of Mathematics, Applied Mathematics and Statistics, Case Western Reserve UniversityCleveland, OH, USA

**Keywords:** spike-response noise entroy, balanced neural networks, information theory, chaos, mutual information

Animals with nervous systems generate complex adaptive behaviors in part through the computational capabilities arising from large networks of interconnected neurons in their brains (Churchland and Sejnowski, 1992). Although a full description of the nervous system would take into account the interactions of central circuits with sensory and motor systems (Chiel and Beer, 1997) it is more common to consider central circuitry in isolation. The individual nerve cells and synaptic junctions that comprise biological neural networks are spatially extended structures with fundamentally stochastic dynamics on a range of spatial and temporal scales (Andersen et al., 2006; Carnevale and Hines, 2006). Nevertheless, much progress has been made in understanding the repertoire of neural behavior through simplified deterministic one dimensional “phase” models such as the Ermentrout-Kopell canonical model (Ermentrout, 1996; Brown et al., 2004; Ermentrout, 2008)^1^.

Even if we restrict attention to isolated networks of deterministic, instantaneously coupled phase models, we confront significant challenges. The behavior of such networks can be chaotic, as evidenced by the divergence of nearby trajectories (positive Lyapunov exponents). If we consider such a “chaotic network” driven by a collection of input signals, it is natural to ask how the intrinsic variability related to the chaotic dynamics impacts the networks' computational capabilities. It is equally natural to view the system as a communications channel. With the input signals drawn from some specified ensemble, and the output taken as the spike trains of (some or all of) the neurons, the mutual information between the input and output ensembles would be of great interest. However, this quantity is difficult to obtain, either analytically or numerically.

In Lajoie et al. (2014), the authors further the analysis of information processing in chaotic deterministic networks by formulating a computationally tractable upper bound on the spike-train noise entropy, building on Monteforte and Wolf (2010) and Lajoie et al. (2013). They study a network of deterministic canonical Ermentrout-Kopell “theta” neurons (Ermentrout and Kopell, 1986) with an *ad-hoc* interaction function. The network connectivity is fixed, sparse and random. Each neuron is driven by a quenched white noise injected current input of the form *I_i_(t)* = η + ϵ *dW_i,t_/dt*. As the authors (and others) have shown previously, the spontaneous activity (i.e., with ϵ = 0) in this class of networks exhibits chaotic behavior. It has been observed that applying an input to such networks (i.e., setting ϵ > 0) can reduce the apparent irregularity of the spike train ensemble. The *spike train entropy* quantifies this reduction in variability; the authors obtain an upper bound on this quantity through a state space partitioning construction that takes advantage of the Kolmogorov-Sinai entropy, which is given in turn by the Lyapunov spectrum, which the authors estimate numerically. They show convincingly that the KS entropy of the spike trains is roughly an order of magnitude smaller than what one would expect from a naive estimate based on the single-cell noise entropy. Their results help make rigorous the observation that the application of a driving stimulus reduces the variability of the resulting spike trains, although the networks remain chaotic.

While this result is a substantive contribution, it is still some steps removed from telling us the mutual information *I(X:Y) = H(Y) − H(Y|X)* between an ensemble of inputs, *X*, and the corresponding ensemble of outputs, *Y*. The authors' result gives a bound on *H(Y|x)* for a specific realization of the frozen noise inputs *x ϵ X*. Because the system is ergodic, this estimate applies as well to the mean entropy *H(Y|X)* [as discussed in Lajoie et al. (2013)]. However, as the authors point out, one cannot replace the entropy *H(Y)* with *H*(*Y*|0), the entropy when the input fluctuations are switched off, since (as they convincingly demonstrate) turning on the input (ϵ > 0) significantly changes the entropy. The entropy that would be needed for calculating the mutual information would be the spike train entropy for the ensemble unconditioned on a specific input—but with an ensemble of different white noises all with fixed ϵ > 0. It would be very interesting if one could investigate how *I*(*X*:*Y*) varied as a function of ϵ; for instance, whether the mutual information changes smoothly or whether there is evidence for some kind of information processing phase transition. The authors' contribution provides a valuable step along the way to a deeper understanding of the impact of chaotic dynamics on computations in deterministic neural networks.

## Conflict of interest statement

The author declares that the research was conducted in the absence of any commercial or financial relationships that could be construed as a potential conflict of interest.

## Footnotes

^1^As pointed out in Wolf et al. (2014), the choice of 1D model can strongly influence the entropy production properties of the resulting network.
